# Platelet-Rich Plasma Therapy: An Effective Approach for Managing Knee Osteoarthritis

**DOI:** 10.7759/cureus.50774

**Published:** 2023-12-19

**Authors:** Jack L Crowley, Varun Soti

**Affiliations:** 1 Orthopedic Surgery, Lake Erie College of Osteopathic Medicine, Elmira, USA; 2 Pharmacology and Therapeutics, Lake Erie College of Osteopathic Medicine, Elmira, USA

**Keywords:** intra-articular knee pathology, knee joint pain, patient outcomes, platelet concentration, knee osteoarthritis (koa), platelet-rich plasma (prp)

## Abstract

Platelet-rich plasma (PRP) is a promising non-invasive therapeutic intervention for knee osteoarthritis (KOA) that has generated significant interest due to anecdotal accounts of its efficacy, resulting in reduced recovery time in various orthopedic interventions. This systematic review examines the effectiveness of PRP in managing KOA. Specifically, it seeks to determine the extent to which PRP can treat KOA patients effectively, alleviate KOA symptoms, and improve patient outcomes. Additionally, the review aims to identify the optimal concentration and composition of PRP required to achieve therapeutic results in KOA. Furthermore, the review investigates whether PRP can modify the synovial environment structurally and immunologically to improve outcomes in KOA patients. We conducted a comprehensive literature search on PubMed, Orthogate, Clinicaltrials.gov, and Embase of clinical trials investigating PRP treatment in KOA patients in the last five years. The results indicated that PRP is effective in treating KOA patients. Evidence shows that PRP therapy can alleviate pain, enhance joint function, increase range of motion, and improve mobility in KOA patients. PRP was effective in treating KOA when the mean platelet concentration of PRP treatment was 4.83 to 5.91 times higher than the baseline whole blood platelet concentration. However, studies investigating PRP with a mean platelet concentration of 3.48 to 4.04 times higher than baseline failed to demonstrate statistically significant improvements. PRP therapy slowed down KOA progression, which validates its effectiveness in impeding further structural damage and arresting the degenerative impact of the disease. Nonetheless, further investigation is necessary to examine how PRP therapy can modify the progression of the disease. Furthermore, future research should identify the most effective platelet concentration levels that provide optimal symptom relief. There is a need for further research to identify the specific PRP configuration that is most pertinent in a clinical setting, as there is a lack of standardization in PRP manufacturing protocols, including the variety of experimental setups and dosing schedules utilized in different studies.

## Introduction and background

Knee osteoarthritis (KOA) is a widespread degenerative joint disease that affects 10% of men and 13% of women 60 or older [1]. As the prevalence of KOA increases with age, effective treatment becomes even more crucial. The impact of moderate to advanced KOA on patients’ daily activities and quality of life is substantial, influencing other potential health concerns. Surgical and non-surgical interventions are currently used for KOA management, with treatment options varying based on individual factors such as activity levels, joint misalignment, and disease progression. Notably, the total knee arthroplasty (TKA) rate has doubled from 1991 to 2010 in the United States and is projected to increase. A significant indication for using TKA for KOA is the failure of non-surgical interventions to halt disease progression or worsening of KOA-related pain. It highlights that non-surgical interventions have yet to provide a genuinely restorative effect on the joint. They merely delay pain onset and allow disease progression to continue [2].

Non-surgical interventions, including hyaluronic acid (HA), prolotherapy (PRL), corticosteroids (CS), and ozone, have been extensively tested for their effects on KOA progression and pain. However, their results have not lived up to the initial promises. HA and CS are commonly used in KOA patients and are widely considered the standard line of treatment for KOA pain management. While these therapies may provide temporary relief, they fail to address the underlying disease process and often have transient effects. Treatments such as CS can cause further joint damage if administered for too long and in high doses, given its immunosuppressant impact [3,4]. 

Platelet-rich plasma (PRP) has evolved as a non-surgical alternative therapy for orthopedic-related interventions, including KOA. PRP involves increasing platelet concentration at the injury site, which is claimed to positively affect the healing process. The increased platelet concentration brings various growth factors that aid in tissue regeneration. PRP’s higher platelet concentration results from separating and isolating blood constituents. PRP is made from an individual patient’s blood, which is centrifuged to isolate a higher concentration of platelets. It is then injected into a joint space or an area of surgical intervention where its supposed therapeutic effects may be elicited on the surrounding tissue. The autologous nature of PRP reduces cost and the risk of adverse reactions. What sets PRP apart is its potential for a bio-restorative impact on the cartilage of KOA patients, reversing cartilage breakdown, fissuring, joint space narrowing, and associated pain [5]. 

This review paper aims to explore the effects of PRP on KOA symptom severity and compare its effectiveness with popular non-surgical interventions currently used in clinical practice. It also delves into the variation in PRP protocols, including platelet concentrations and types, to aid researchers in standardizing PRP as an evidence-based, quality-controlled therapy. It aims to address the following key questions: 1. Can PRP be an effective modality in treating KOA patients? 2. To what extent can PRP alleviate KOA symptoms and improve patient outcomes? 3. What is PRP’s optimal concentration and composition for achieving therapeutic results in KOA? 4. Does PRP alter the synovial environment structurally and immunologically to enhance outcomes in KOA patients?

By addressing the questions mentioned above, this paper sheds light on the potential role of PRP as an emerging, non-invasive treatment option for KOA.

## Review

Data sources and study selection

**Figure 1:**
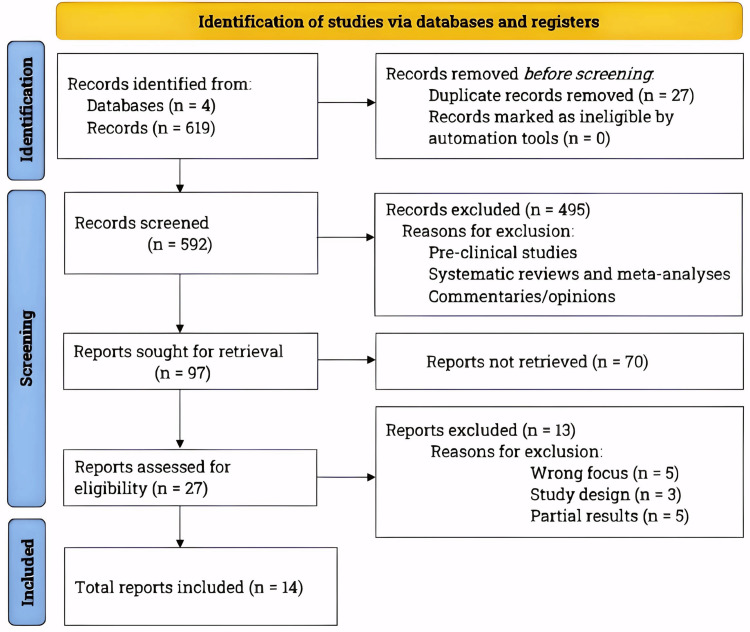
The PRISMA flowchart showing literature search and study selection process. Conforming to the PRISMA guidelines, we conducted an extensive literature search utilizing databases, including PubMed, Orthogate, Embase, and Clinicaltrials.gov. The inclusion criteria involved clinical trials that examined the effects of Platelet-Rich Plasma on knee osteoarthritis patients and were published within the last five years. ﻿n, number of studies; PRISMA, Preferred Reporting Items for Systematic Reviews and Meta-Analyses.

Following the Preferred Reporting Items for Systematic Reviews and Meta-Analyses (PRISMA) guidelines, we performed a comprehensive literature search from January to July 2023 on PubMed, Orthogate, Embase, and Clinicaltrials.gov. Please refer to Figure 1 for the PRISMA flowchart depicting the literature search and study selection [6].

Our search term "PRP and Knee Osteoarthritis" targeted clinical trials that compared PRP with non-surgical treatments and control groups in the last five years. To determine the level of clinical evidence, we consulted previous literature [7]. Only studies meeting our inclusion criteria with a clinical evidence level of II or higher and published in English were included in our analysis (Table 1).

**Table 1:** Criteria for selecting studies. In this review, we have considered studies investigating the effects of Platelet-Rich Plasma treatment on patients with knee osteoarthritis. Our inclusion criteria were limited to studies published in English and those that met the specifications outlined in this table.

Inclusion Criteria	Exclusion Criteria
Randomized clinical trials	Observational clinical studies
Placebo-controlled clinical trials	Cross-sectional clinical studies
Single-blinded clinical trials	Retrospective clinical studies
Double-blinded clinical trials	Case Reports
Non-blinded clinical trials	Systematic reviews
Pilot clinical studies	Meta analyses
	Narrative reviews
	Commentaries/opinions
	Preclinical studies

KOA: pathophysiology, classification, and grading

KOA represents the primary cause of arthritis and develops due to gradual cartilage breakdown and joint space thinning over time. This process involves various factors, such as inflammatory mechanisms, increased mechanical stress [possibly due to a high body mass index (BMI)], excessive physical activity, metabolic disorders, and genetic predisposition. The key constituents of cartilage include water, chondrocytes, proteoglycans, and type II collagen. In KOA, the inflammatory and mechanical stresses culminate, leading to altered ratios of these components and disrupted collagen formation. Consequently, the cartilage loses its elasticity, resulting in tissue degradation and erosion of the articular surface. As the disease progresses, chondrocytes often enlarge, accompanied by an imbalance between degradative enzymes and their inhibitors within the joint space [8].

KOA can be categorized into two types: primary osteoarthritis, arising without trauma and commonly linked to advanced age, and secondary osteoarthritis, caused by joint misalignment or physical injury. Diagnostic confirmation of KOA necessitates radiographic evaluation, with the affected joint displaying characteristic signs of degeneration that adhere to the Kellgren-Lawrence grading scale. This widely accepted scale defines different stages of disease severity, ranging from grade 0 to 4. The classification is based on the presence and size of osteophytes, the degree of joint space narrowing, and the extent of cartilage sclerosis as observed on imaging. Radiographic findings for grade 0 indicate the absence of osteophyte changes, while grade 4 severity encompasses large osteophytes, significant joint space reduction, sclerosis, and bone deformity [9].

PRP: classification and protocol

PRP can be classified as either activated or non-activated, as well as leukocyte-rich or leukocyte-poor [10]. Once the patient’s blood is centrifuged, serum samples are extracted from just above the area of high leukocyte concentration called the buffy coat, where platelet concentration is at its highest [11]. The process of centrifugation, including time, speed, and number of rounds, varies in each PRP protocol. Until a standardized approach is established, this remains a significant issue in comparing different clinical trials. Beyond their well-known role in clot formation, platelets play a crucial role in hemostasis and cell signaling. They contain various growth factors and biologically active molecules in their alpha and dense granules [12].

Increasing platelet concentration in PRP also increases the levels of growth factors and cytokines, potentially leading to the proposed therapeutic effects. Activated PRP involves using calcium chloride or autologous thrombin to induce platelet granule release, activation, and aggregation [13]. On the other hand, non-activated PRP allows platelets to be activated through exposure to tissue factors and the extrinsic platelet activation mechanism. Studies have shown that non-activated PRP is more associated with cartilage regeneration and bone formation [14].

To be considered leukocyte-rich PRP (LR-PRP), a PRP preparation must have a higher leukocyte concentration than the patient’s baseline whole-blood leukocyte concentration [15]. LR-PRP has demonstrated pro-inflammatory and catabolic effects in certain studies, unlike leukocyte-poor PRP (LP-PRP). However, the clinical implications of choosing between LR-PRP and LP-PRP have yet to be fully explored in human clinical trials, highlighting the challenge of standardizing PRP as a treatment. Many studies, including those reviewed here, fail to mention the classification of the PRP protocol used, underscoring the need for improved research quality to determine the clinical significance of PRP and establish standardized protocols [12].

Compelling clinical evidence of PRP’s effectiveness in treating KOA

A randomized controlled trial conducted by Raeissadat et al. (2021) examined the effectiveness of PRP in managing knee osteoarthritis compared to plasma rich in growth factor (PRGF), HA, and ozone. The study comprised 200 patients aged between 50 and 75 diagnosed with primary KOA with a Kellgren-Lawrence grade 2 or higher. All patients had experienced KOA-related pain and symptoms for at least three months. The researchers randomized the patients into four groups: PRP, PRGF, HA, and ozone [16].

In the PRP group, patients received two doses of 2 milliliters (mL) of non-activated LR-PRP at a three-week interval. The PRGF group followed an identical dosing schedule as the PRP group. The HA group received three weekly doses of HA, and the ozone group received ozone according to the same dosing schedule as the HA group. The study assessed knee pain and function using the Western Ontario and McMaster Universities Arthritis Index (WOMAC) scale, Visual Analog Scale (VAS), and the Lequesne Algofunctional (LEQ) index. The evaluation occurred at baseline (pre-treatment) and two, six, and 12 months after treatment [16].

The intergroup analysis focusing on the PRP group revealed significantly lower VAS scores compared to the ozone group at six months (p < 0.05) and lower scores than the HA and ozone groups at 12 months (p < 0.05). Additionally, the WOMAC analysis demonstrated a substantial decrease in WOMAC scores with PRP treatment compared to ozone treatment at the six-month follow-up (p < 0.05), as well as the HA and ozone treatments at the 12-month follow-up (p < 0.05). Furthermore, at the six-month and 12-month follow-ups, there were notable differences in LEQ indices between the PRP group and the ozone group (p < 0.05), as well as between the PRP group and the HA and ozone groups (p < 0.05). Notably, while the study compared PRP therapy to other widely regarded effective interventions, there was no control or placebo group [16].

Bansal et al. (2021) conducted a randomized controlled trial to investigate the effectiveness of PRP in treating KOA patients. They compared PRP to HA therapy and aimed to standardize the PRP protocol for optimal therapeutic effects. The study included 132 patients aged 50 years or older who had experienced pain or swelling for at least three months and met the clinical and radiological criteria for grade I-II KOA, according to the American College of Rheumatology [17].

The study randomized patients into two groups: the PRP group and the HA group. Patients in the PRP group received a dose of 8 mL of non-activated LP-PRP. In the HA group, patients received a single dose of 4 mL of high molecular weight HA. The researchers estimated knee pain and function based on WOMAC sores, International Knee Diagnostic Committee (IKDC) scores, 6-minute walking distance (6MWD) results, joint space width (JSW) (assessed through radiographic imaging), and articular cartilage thickness [gauged by magnetic resonance imaging (MRI)]. They assessed patients at baseline (pre-treatment) and one, three, six, nine, and 12 months post-treatment [17]. 

The study results showed that WOMAC scores were significantly lower in the PRP group compared to the HA group (p < 0.05) at all study points for follow-up. Also remarkable were IKDC scores at three-month follow-up (p < 0.05) and onward (p < 0.05). Patients in the PRP group had a statistically significant increase in IKDC scores (p < 0.05) and substantially improved joint function and activity levels compared to the HA group. There was also a notable increase in the 6MWD test results for patients in the PRP group than the HA group (p < 0.05). Interestingly, JSW decreased in both groups, suggesting disease progression. However, the two treatment groups concerning JSW had no statistically significant difference. Another impressive finding was the maintenance of cartilage thickness in the PRP group. The number of patients who maintained cartilage thickness throughout the year-long study follow-ups was significantly higher in the PRP group than in the HA group (p < 0.05) [17]. 

These findings indicated that PRP provided effective pain relief to KOA patients and exhibited cartilage thickening effects compared to HA therapy. A potential limitation of this clinical trial was the absence of control to evaluate placebo effects. Nonetheless, the researchers corroborated the findings of Raeissadat et al. (2021) [16] and attempted to standardize the PRP protocol, which was a pioneering endeavor. They utilized PRP based on the number of platelets per PRP sample rather than a fixed platelet concentration higher than the patient’s whole-blood platelet concentration [17].

In a randomized controlled trial, Dulic et al. (2021) compared the effectiveness of three treatments, PRP, bone marrow aspirate concentrate (BMAC), and HA, in treating patients with KOA. The study included 175 KOA patients aged 18 years and older, with a Kellgren-Lawrence grade of 2-4, and symptomatic within the past 12 months. These patients were randomly assigned to one of three groups: the PRP, BMAC, or HA groups. Per the treatment protocols, patients in the PRP group received a single dose of an unstated volume of non-activated LR-PRP (the platelet concentration was 7.23x baseline, and the leukocyte concentration was 2.2x baseline); patients in the HA group were given a weekly injection of 2 mL sodium hyaluronate for three weeks; and patients in the BMAC group received a single intra-articular injection of 5-6 mL autologous BMAC. The primary outcome measures were knee pain and function assessed by VAS, WOMAC, IKDC, and Knee Injury and Osteoarthritis Outcome Score (KOOS). The study evaluated patients at baseline and at different time points after the treatment: VAS scores were assessed at three, seven, 14, and 21 days post-injection; WOMAC, IKDC, and KOOS scores were evaluated at one, three, six, nine, and 12 months post-injection [18]. 

The study results demonstrated significant improvement in knee pain and function across all groups compared to baseline. Specifically, PRP substantially alleviated knee pain, as shown by reduced VAS (p < 0.001) and WOMAC scores (p = 0,001) compared to baseline. PRP also resulted in significant improvement in IKDC (p < 0.001) and KOOS scores (p < 0.01) compared to baseline. However, the intergroup analysis of VAS scores demonstrated that BMAC consistently outperformed the HA and PRP groups at each follow-up time point (p < 0.001). At the same time, there was no significant difference in VAS scores between the HA and PRP groups. Regarding the KOOS overall analysis, BMAC significantly improved knee function compared to the HA group at all follow-ups, and it showed significant improvements compared to the PRP group between one (p < 0.001) and three months (p = 0.004). Nonetheless, there was no significant difference in knee function between the HA and PRP groups at the post-treatment follow-ups [18]. 

The analysis of WOMAC scores revealed that BMAC remarkably reduced joint pain and stiffness compared to the HA group (p < 0.035), with no significant difference compared to the PRP group. The IKDC score analysis further indicated that BMAC significantly increased IKDC scores compared to the HA group on follow-ups. While the BMAC group had higher IKDC scores than the PRP group at follow-ups, the differences were only statistically significant at the three-month (p < 0.002) and six-month (p < 0.033) follow-ups. Notably, the absence of a control group limited our ability to account for potential placebo effects. Furthermore, the relatively high platelet concentration utilized in the study could amplify PRP’s effectiveness. However, it is crucial to consider that differences in the preparation protocol for PRP may introduce confounding factors when comparing patient outcomes across varying platelet concentrations [18].

In a randomized controlled clinical trial, Rahimzadeh et al. (2018) compared the effectiveness of PRP and PRL in patients with KOA. They recruited 42 individuals diagnosed with KOA, aged between 40 and 70, with a Kellgren-Lawrence grade of 1-2. The researchers randomly assigned these participants into two groups: the PRP group and the PRL group. The study’s dosing protocol specified that patients in the PRP group received two doses of 7 mL of PRP a month apart. In contrast, patients in the PRL group received a 7 mL injection of 25% dextrose following the same dosing schedule. However, the study did not specify the type of PRP administered, whether it was activated or non-activated, and the leukocyte concentration [19].

The study's primary outcomes were knee pain and function, as evaluated by WOMAC scores. The researchers evaluated patients at their baseline just before treatment. Post-treatment, patients were assessed at one, two, and six months. When comparing the results between the two treatment groups, the PRP group had significantly lower WOMAC scores at the two- and six-month follow-ups, indicating reduced pain and improved function, with p-values of 0.004 and 0.009, respectively [19].

These findings demonstrated the effectiveness of PRP in providing pain relief and joint function in patients with KOA, showing its superiority over PRL, an 80-year-old therapy used for pain control in KOA. While PRL may have been a popular approach, its efficacy is still being determined due to its short-lasting effects and the need for multiple injections over an extended period. Consequently, further clinical trials with a larger sample size are necessary to determine the benefits and effectiveness of PRP compared to PRL in patients with KOA [19].

In a randomized controlled clinical trial, Sdeek et al. (2021) assessed the effectiveness of PRP and HA in managing KOA. They recruited 189 patients diagnosed with primary KOA with a Kellgren-Lawrence score of 2-3. All patients had been experiencing chronic knee pain for at least six months. The patients were randomly assigned to two groups: the PRP and HA groups. The protocol required the PRP group to receive three doses of 2.5 mL non-activated LP-PRP once every two weeks and 2.5 mL of high molecular weight HA with the same dosing schedule. Knee pain and function were assessed using WOMAC, IKDC, and VAS scores. Follow-up evaluations were conducted at two, six, 12, 18, 24, 30, and 36 months post-injection [20].

The study's results demonstrate that, at the 36-month follow-up, patients who received PRP injections had significantly lower VAS scores than those who received HA injections. The average VAS score in the PRP group was 40.9, whereas it was 60.3 in the HA group. The trend was also observed in the WOMAC scores, as the PRP group exhibited lower scores, with an average score of 60.6, compared to the HA group, with an average score of 64.2, at the 36-month follow-up. Analysis of the IKDC scores revealed that the PRP group had a prolonged period of improvement, with an average score of 55.2, compared to the HA group, with an average score of 46.1, at the 36-month follow-up. However, the researchers did not report the statistical comparison [20].

The study outcomes indicated that PRP was considerably more effective than HA in reducing knee pain and improving joint function. Notably, the PRP used in this study had a higher platelet concentration than many other studies. As a result, the study's design included long-term follow-up evaluations of up to 36 months, demonstrating PRP treatment's sustained beneficial effects. This discrepancy in concentration may have affected the study's results, highlighting the considerable variation in PRP preparation protocols. Higher platelet concentrations may result in more clinically significant effects within the joint space than lower concentrations [20].

In a randomized controlled clinical trial, Wu et al. (2018) compared the effectiveness of PRP in pain management and maintaining muscle strength to placebo in bilateral KOA patients. They recruited 20 patients (40 knees) diagnosed with primary bilateral KOA of the same grade, an Ahlback Stage I-II, and between 40-70 years of age. All patients presented with bilateral pain with at least a VAS score 4. The researchers randomized these patients into the PRP and saline control (placebo) groups, with patients in the PRP group receiving a single dose of 4 mL non-activated LR-PRP and the placebo group receiving a single dose of 4 mL saline injection. The study evaluated knee pain and function using WOMAC scores and assessed muscle strength through flexion and extension. The researchers conducted assessments at baseline, pre-injection, and at two weeks, one month, three months, and six months post-injection [21].

The statistical analysis of the study findings indicated a significant decrease in WOMAC scores, representing reduced pain and improved knee function, in the PRP group compared to the placebo group at the follow-up appointment (p = 0.01 at two weeks, p < 0.05 at one month, p = 0.001 at three months, p < 0.05 at six months post-injection). However, the two groups had no significant difference regarding muscle strength during flexion and extension. It is worth noting that the PRP protocol did not specify the platelet concentration used. Additionally, the unique feature of this study was the inclusion of patients with bilateral KOA who received bilateral PRP knee injections, distinguishing it from other study designs [21].

In a randomized controlled clinical trial, Nunes-Tamashiro et al. (2020) compared PRP with triamcinolone hexacetonide (TH), a glucocorticoid corticosteroid, and a placebo. The objective was to assess their efficacy in providing pain relief and improving joint function, range of motion (ROM), and mobility for patients with KOA. The study enrolled 100 patients aged 40-85 diagnosed with primary KOA. Participants had a radiographically confirmed Kellgren-Lawrence grade of 2-3 and a minimum pain duration of three months. Randomization assigned patients to the PRP, TH, and placebo groups. The PRP group received a single dose of an unstated volume of non-activated PRP, while the TH and placebo groups received 2 mL of TH and saline injections, respectively [22].

The research team evaluated knee pain function, structural damage, and disease progression using various assessments, including VAS, WOMAC, 6MWD, time to complete the up-and-go test, and ROM in flexion and extension. Radiographic examination assessed the disease progression. Assessments occurred at baseline (pre-treatment) and four, eight, 12, and 52 weeks post-treatment [22]. 

The study findings showed significant improvements in the PRP and TH groups compared to the placebo group. Both PRP and TH groups experienced notably reduced VAS scores (p < 0.05), decreased WOMAC scores (p < 0.05), and improved outcomes in 6MWD (p < 0.05), time to complete the up-and-go test (p < 0.05), and ROM in flexion and extension (p < 0.05). However, the most noteworthy finding was that patients in the PRP group exhibited no statistically significant disease progression from baseline to the 52-week follow-up post-treatment compared to the placebo and TH groups. It demonstrates the effectiveness of PRP in slowing down knee deterioration and preventing further structural damage [22].

It must be stated that the PRP protocol used in the study did not account for leukocyte count. Consequently, it remains unknown whether the PRP used was leukocyte-rich or leukocyte-poor. Nevertheless, there were no indications that additional steps were taken to increase the leukocyte count, leading one to assume it was probably LP-PRP. Nonetheless, the study showed the protective effect of PRP in KOA, thus paving the way for further investigation of the underlying mechanisms through which PRP can halt disease progression within the intra-articular space and prevent structural damage in KOA [22].

In a randomized controlled clinical trial, Huang et al. (2019) examined the effects of PRP, HA, and CS on joint pain and functional deficits observed in patients with KOA. They enrolled 120 KOA patients aged 40-65 with a Kellgren-Lawrence grade of 1-2. The researchers divided patients into the PRP, HA, and CS groups through randomization. The PRP group received a single dose of 4 mL LP-PRP once a week for three weeks, while the HA group received a single dose of 2 mL HA once a week for three weeks. Patients in the CS group received a single 1 mL dose of unspecified CS. The researchers evaluated the participants for knee pain and function using VAS and WOMAC scores before and at three, six, nine, and 12 months after the treatment [23].

The study findings revealed a significant reduction in WOMAC scores for all groups at follow-up compared to baseline scores. Interestingly, the intergroup analysis demonstrated that the PRP group exhibited significantly lower WOMAC scores than both the HA group (p < 0.05) and the CS group (p < 0.05) at the various study follow-up time points. This suggests that PRP is more effective than other treatments in managing KOA patients. Moreover, a statistically significant improvement in VAS scores was observed across all study time points compared to the baseline, with p < 0.05. However, no intergroup analysis was conducted to evaluate post-treatment differences in VAS pain scores [23].

Interestingly, this study excluded patients with a BMI of 30 or higher because a higher BMI directly correlates with increased physical stress on the joint during similar activity levels. Additionally, the platelet concentration used in this study was around two times that is found in whole blood, which, despite being on the lower end, presents an intriguing comparison to other studies with similar treatment groups and outcome measures [23]. 

Table 2 shows a succinct summary of studies demonstrating compelling clinical evidence of PRP efficacy in treating KOA.

**Table 2:** Platelet-rich-plasma (PRP): Key studies showing compelling evidence of PRP’s effectiveness in knee osteoarthritis (KOA). The table presents a compilation of critical studies that showcase the essential study designs and findings demonstrating PRP's efficacy in treating patients with KOA. These studies are clinical trials that have successfully met the criteria for inclusion in this review and carry a clinical evidence level of II or higher. 6-MWD, 6-Minute Walking Distance; BMAC, Bone Marrow Aspirate Concentrate; HA, Hyaluronic Acid; IKDC, International Knee Documentation Committee; JSW, Joint Space Width; KOOS, Knee Injury and Osteoarthritis Outcome Score; LEQ Index, Lequesne Algofunctional Index; mL, Milliliters; MRI, Magnetic Resonance Imaging; PRGF, Plasma Rich in Growth Factor; PRL, Prolotherapy; p, Probability; ROM, Range of Motion; TH, Triamcinolone Hexacetonide; VAS, Visual Analogue Scale; vs., Versus; WOMAC, Western Ontario and McMaster Universities Osteoarthritis Index.

Authors	Sample Size	Age (Years)	Dose/Platelet Concentration	PRP Type	Treatment Groups	Assessment Time	Assessment Tools	Findings
Raeissadat et al. (2021) [16]	200 patients	50-75	2 mL dose weekly for 3 weeks at 4-6x the whole blood	Non-activated and leukocyte-rich	HA (49) vs. PRP (52) vs. PRGF (51) vs. Ozone (48)	Baseline, 2, 6, and 12 months	WOMAC, VAS, and LEQ Index	PRP treatment led to lower VAS scores at six months when compared to ozone therapy, and at 12 months when compared to both hyaluronic acid (HA) and ozone therapy (p < 0.05). A similar significant decrease trend was observed with PRP in the WOMAC and LEQ indices as well (p < 0.05).
Bansal et al. (2021) [17]	132 patients	50 or older	Single 8 mL at 2.78-8.33x the whole blood (standardized to 10 billion platelets/8 mL injection)	Non-activated and leukocyte-poor	PRP (64) vs. HA (68)	Baseline, 1, 3, 6, and 12 months	WOMAC, IKDC, 6-MWD, JSW (via X-ray), and Articular cartilage thickness (via MRI)	PRP treatment resulted in reduced WOMAC scores across all time points (p < 0.05), increased IKDC scores from the three-month mark onwards (p < 0.05), and augmented 6-MWD (p < 0.05). PRP therapy was associated with decreased cartilage loss (p < 0.05). However, no significant differences were observed in JSW changes between the groups.
Dulic et al. (2021) [18]	175 patients	18 or older	Single injection at 7.23x the whole blood and leukocyte conc. at 2.22x the whole blood (Dose unspecified)	Non-activated and leukocyte-rich	BMAC (111) vs. PRP (34) vs. HA (30)	Baseline, IKDC + WOMAC + KOOS (1, 3, 6, 9, and 12 months), VAS (3 days after, 7 days after, 14 days after, and 21 days after)	VAS, WOMAC, IKDC, and KOOS	PRP substantially alleviated knee pain, as shown by reduced VAS (p < 0.001) and WOMAC scores (p = 0,001) compared to baseline. PRP resulted in significant improvement in IKDC (p < 0.001) and KOOS scores (p < 0.01) compared to baseline. There was no significant difference in VAS scores between the HA and PRP groups. BMAC outperformed all groups across the parameters assessed.
Rahimzadeh et al. (2018) [19]	42 patients	40-70	7 mL dose monthly for 2 months at 5x the whole blood	Not stated	PRP (21) vs. PRL (21)	Baseline, 1 month, 2 months, and 6 months	WOMAC	PRP treatment significantly decreased WOMAC scores at two and six months compared to PRL (p = 0.004, p = 0.009, respectively).
Sdeek et al. (2021) [20]	189 patients	45-65	Unspecified dose at two-week interval for 6 weeks at 8.2x the whole blood	Non-activated and leukocyte-poor	PRP (95) vs. HA (94)	Baseline, 2, 6, 12, 18, 24, 30, and 36 months	WOMAC, IKDC, and VAS	PRP treatment showed significant improvement in WOMAC, IKDC, and VAS scores compared to baseline scores. However, no statistical comparison was conducted.
Wu et al. (2018) [21]	20 (40 knees) patients	50-75	Single injection of unstated concentration	Non-activated and leukocyte-rich	PRP (20) vs. Saline (20)	Baseline, 2 weeks, 1 month, 3 months, and 6 months	WOMAC, Knee Flexion and Extension Strength	PRP treatment significantly reduced WOMAC scores compared to the control (saline) group. The results were consistent across multiple follow-up assessments at two-week, one-month, three-month, and six-month intervals (p = 0.01, p = 0.05, p = 0.001, p = 0.05, respectively).
Nunes-Tamashiroet al. (2022) [22]	100 patients	40-85	Single injection at 4.61x whole blood	Non-activated (leukocytes not measured)	PRP (34) vs. TH (33) vs. Saline (33)	Baseline, 4 weeks, 8 weeks, 12 weeks, and 52 weeks	VAS, 6MWD, WOMAC, Quality of life, Time to up-and-go test, ROM Flexion/Extension, and Radiographic Assessment	Compared to the placebo (saline) group, patients in both PRP and TH groups experienced notably reduced VAS scores (p < 0.05), decreased WOMAC scores (p < 0.05), and improved outcomes in 6MWD (p < 0.05), time to complete the up-and-go test (p < 0.05), and ROM in flexion and extension (p < 0.05). Most noteworthy was that patients in the PRP group exhibited no statistically significant disease progression (p = 0.311) from baseline to the 52-week follow-up post-treatment compared to the placebo and TH groups.
Huang et al. (2019) [23]	120 patients	40-65	Single dose at 2x the whole blood	Non-activated and leukocyte-poor	PRP (40) vs. HA (40) vs. Corticosteroids (CS) (40)	Baseline, 3 months, 6 months, 9 months, and 12 months	WOMAC and VAS	PRP treatment decreased WOMAC compared to other groups (p < 0.05). PRP significantly decreased VAS compared to baseline at all time points (p < 0.05), but no intergroup analysis was done.

Limited or inconclusive evidence of PRP’s effectiveness in treating KOA

Bennell et al. (2021) conducted a randomized clinical trial to assess the effectiveness of PRP in managing KOA and restoring tibial cartilage volume. The study involved 288 patients diagnosed with primary KOA, each with a pain score of 4 or greater on an 11-point scale. All patients were 50 years of age or older and had a radiographically confirmed history of tibiofemoral osteoarthritis. These patients were randomly assigned to the PRP or saline control groups. The PRP group received intra-articular injections of non-activated LP-PRP in three doses of 5 mL each at weekly intervals. In contrast, the control group received saline injections with the same dosing schedule [24]. 

To assess the knee pain and cartilage loss, the researchers used an 11-point pain scale and MRI for monitoring medial tibial cartilage volume. They conducted assessments at the baseline (just before the treatment administration) and after 12 months. Although the difference between groups in the pain scores was not statistically significant (p = 0.17), the PRP group reported lower pain scores. Moreover, no significant difference (p = 0.81) was found in the annual change of medial tibial cartilage volume, although the placebo group had a more minor loss of cartilage [24].

This study compared the efficacy of saline and PRP injections for KOA patients. While other studies have compared PRP to other proven therapies, this was a more direct comparison. However, if the study had used a standard scale of knee pain and function, such as the WOMAC score, it would have been easier to compare its results to those of other studies [24].

In a randomized clinical trial, Dório et al. (2021) explored the efficacy of intra-articular PRP treatment compared to autologous plasma and saline injections. They recruited 62 patients diagnosed with primary KOA with a Kellgren-Lawrence grade of II-III while being symptomatic in the past week and at the ages of 45-80 years old. The researchers randomized these patients into three groups: the PRP group, the plasma group, and the saline control group. The study’s dosage protocol required patients in the PRP group to receive two doses of 1.4-5 mL of non-activated LP-PRP at a two-week interval. The volume varied between patients to maintain a consistent platelet concentration compared to the patient's baseline whole blood platelet concentration. The plasma and saline groups also received two 1.4-5 mL doses with a two-week interval dependent on the patient’s whole blood platelet concentration [25]. 

The study evaluated knee pain and function via VAS, WOMAC, KOOS, and Outcome Measures in Rheumatology-Osteoarthritis Research Society International response criteria. The study protocol assessed the patients at baseline just before treatment administration and again at six weeks, 12 weeks, and 24 weeks post-treatment. All three groups had a significant decrease from baseline on VAS. However, in terms of all of the intergroup analyses in the study, only a single subsection of the KOOS scale, sport/recreation 0-100, showed a significant decrease in the PRP group compared to the plasma and saline groups (p = 0.005) [25].

This study examined the pain relief and joint function effects of PRP versus plasma with saline as a control. This is the first study to compare PRP to the potential impact that plasma containing only naturally occurring levels of platelets may have. This helps to directly correlate the increased platelet count within the PRP to any effects seen after the study [25].

In a randomized clinical trial, Küçükakkaş et al. (2022) compared the effectiveness of PRP and HA on pain management and maintenance of femoral cartilage thickness in KOA patients. They recruited 40 patients diagnosed with primary KOA of a Kellgren-Lawrence grade II-III via radiographic evidence and were between 18-80 years of age. The researchers randomized these patients into two groups: the PRP group and the HA group. The study’s dosage protocol required patients in the PRP group to receive a 5 mL dose of non-activated LP-PRP. On the other hand, patients in the HA group received a single injection of high molecular weight HA [26]. 

Knee pain, function, and structural damage were assessed using the VAS at rest and during movement, the WOMAC test, and femoral cartilage thickness evaluation. The study protocol required patients to undergo assessment at baseline and again at one-month and six-month intervals. Both experimental groups demonstrated a statistically significant improvement in pain reduction (p < 0.05) and functional improvement (p < 0.05) from baseline to study completion as measured by all three tests. However, the two groups had no significant difference (p > 0.05). A significant increase in cartilage regeneration was observed in the HA group compared to the PRP group as measured by femoral cartilage thickness in the medial, lateral, and mean cartilage (p = 0.003, p < 0.001, p < 0.001, respectively). In contrast, the PRP group had no significant increase in cartilage thickness (p > 0.05) [26]. 

In this study, PRP injections were compared to HA injections. However, there was no control group in the study. The concentration of platelets in the PRP protocol used was relatively high. Yet, the study found more clinically relevant outcomes with HA injections, which is notable. However, this study had a significantly smaller sample size than other studies, demonstrating that PRP was more effective than HA. While pain relief is an important outcome, especially for the patient's quality of life, measurable structural changes that can reverse disease progression are considered the gold standard of disease treatment [26]. 

In a randomized clinical trial, Pishgahi et al. (2020) compared the effects of dextrose PRL, PRP, and autologous conditioned serum (ACS) on pain management and joint function in KOA patients. They recruited 92 patients diagnosed with primary KOA with a radiographically verifiable Kellgren-Lawrence grade of II-IV with no specific age restrictions. The researchers randomized these patients into three groups: the PRP group, the PRL group, and the ACS group. According to the study’s protocol, patients in the PR group received a non-activated LP-PRP injection of 5 mL once a week for three weeks. Meanwhile, the PRL and ACS groups were given an injection of 5 mL and 2 mL once a week for two weeks [27].

Knee pain and function were evaluated using the VAS and the WOMAC test. The patients were assessed at their baseline and then retested at the one-month and six-month time frames. The results showed that the PRL group did not experience a significant decrease in VAS scores throughout the study (p > 0.05). The PRP group had a significant reduction in scores only at the one-month time frame (p = 0.019) when compared to the baseline, while the ACS group had a significant decrease in scores for both time frames (p = 0.011 and p < 0.001, respectively). In the intergroup analysis, the PRP treatment did not show a significant decrease in scores compared to PRL, although scores were generally lower. The ACS group had consistently lower scores than the PRP and PRL groups. This decrease showed statistical significance when compared to all of the PRL group's scores (p = 0.044, p < 0.001) while only being significant when compared to the PRP group at the six-month time frame (p < 0.001) [27].

Regarding the WOMAC test, the PRL group did not experience a significant decrease in scores for all time frames, while both the PRP and ACS groups showed no significant changes at the one-month time frame, with scores eventually reaching a statistically significant decrease by the six-month time frame (p = 0.037 and p < 0.001, respectively). The intergroup analysis showed that the PRP and ACS groups significantly decreased scores compared to the PRL group at all time frames (p < 0.001 for PRP and ACS at both time frames). At the same time, there was no significant difference between the PRP and ACS groups in all time frames [27].

In this study, the efficacy of PRP and ACS injections, separately, were compared against traditional PRL injections for patients diagnosed with KOA. Notably, the study compared two relatively new, non-invasive approaches (PRP and ACS) against the classic PRL injection approach used for pain relief in KOA patients for most of the past century. It is worth noting that this is the only study we reviewed with no age limit; the only inclusion factor was knee pain and lack of function related to radiologically confirmed grade II-IV KOA [27].

The study conducted by Tucker et al. (2021) compared the effects of PRP and saline on joint pain and function in patients diagnosed with primary KOA. The study focused on environmental changes within the synovial space. Sixteen veterans, aged 40 or above with a Kellgren-Lawrence grade of II-III, were recruited and randomly assigned to either the PRP or the saline control group. The study followed a dosage protocol in which patients in each group were administered a 5 mL dose of either PRP or saline [28]. 

The VAS, WOMAC, synovial fluid, and radiographic assessments were used to evaluate knee pain function, structural damage, disease progression, and synovial fluid changes. The baseline assessment was followed by four subsequent evaluations at 14 weeks, three, six, and 12 months post-treatment, with radiographic imaging only at six months post-treatment. The WOMAC analysis included three scores for pain, stiffness, and physical function [28]. 

The PRP treatment group significantly reduced stiffness at three (p < 0.05), six (p < 0.05), and 12 months (p < 0.05), and reduced physical function impediments at three (p < 0.05) and six months (p < 0.05). Although the PRP group experienced reduced pain as they had consistently decreased VAS scores, the reduction was not statistically significant. No intergroup analysis was performed. In the PRP treatment group, a substantial increase in the alpha-2-macroglobulin (A2M) protein was observed within the synovial fluid (p < 0.005). A2M has been shown to enhance prothrombin activation by inhibiting the protein C/protein S system in the coagulation cascade [28]. 

The synovial fluid analysis revealed variations in the cytokine profile, proteolytic enzymes, extracellular matrix (ECM) proteins, their inhibitors, and angiogenic response factors. The PRP treatment group showed a moderate increase in interleukin 5, 6, 8, 10, and tumor necrosis factor-alpha of the cytokine profile, but no statistical analysis was performed on the other subgroups. The radiographic examination could not draw considerable conclusions due to the small sample size [28]. 

The study highlighted the effectiveness of PRP as a treatment for KOA, as demonstrated by a reduction in pain, stiffness, and physical function impediments. The study's results also shed light on the changes within the synovial fluid after treatment. The synovial fluid analysis focused on measuring inflammatory cytokines, various growth factors, changes in mesenchymal stem cell and synoviocyte gene expression, and variation in ECM/angiogenic/intercellular adhesion biomarkers between the different treatment groups. This quantitative analysis provides a benchmark for further investigation into the pathways through which PRP may elicit a structurally restorative effect on the knee. However, the study had a small sample size, limiting the findings' generalizability [28]. 

Additionally, the analysis of cytokines and biomarkers only focused on the early phase response (two weeks after treatment). Thus, long-term clinically relevant changes to the knee synovial environment may have been missed. This study highlights the potential of PRP as a treatment for KOA and emphasizes the need for further research to fully understand the mechanisms and long-term effects of PRP treatment [28].

Lewis et al. (2022) conducted a randomized clinical trial to assess the efficacy of LP-PRP in treating symptomatic early tibiofemoral osteoarthritis. The study included 102 patients diagnosed with early radiological evidence of KOA with a Kellgren-Lawrence grade of 0-II, aged 18 or older, and experiencing pain for at least four months. The researchers divided these patients into three groups: the single-dose PRP group, the full-dose PRP group, and the saline control group [29].

The study's treatment protocol required patients in the full-dose PRP group to receive a single 4-6 mL LP-PRP injection weekly for three weeks. The single-dose PRP group was given a 4-6 mL dose of LP-PRP, followed by two 5 mL doses at weekly intervals for three weeks. The saline group received a single 5 mL dose of saline at weekly intervals for three weeks. The researchers evaluated knee pain function and structural damage using KOOS, VAS, and EuroQol five-dimension five-level index (EQ-5D-5L). The patients' baseline was assessed just before treatment, and the assessment schedule was repeated at six weeks, 12 weeks, 26 weeks, and 52 weeks post-treatment [29].

In the KOOS analysis, the saline and single-dose PRP groups significantly decreased scores throughout the study (p < 0.0041). Meanwhile, the full-dose PRP group only achieved statistical significance in the final measurement at 52 weeks (p = 0.007). The intergroup analysis only revealed a substantial decrease in scores at a single time frame, 12 weeks, and only within the full-dose PRP group. For the intragroup analysis of the EQ-5D-5L test, the saline group showed a significant decrease in scores at the 12-week measurement (p = 0.036). The single-dose PRP group recorded a significant decline in scores at the six-, 12-, and 26-week measurements (p = 0.036), while the full-dose PRP group had no significant effect on scores throughout the study [29].

Intergroup analysis of the EQ-5D-5L showed insignificant differences between both PRP groups and the saline control group throughout the study. The statistical analysis in this study did not compare to the baseline; instead, it measured the significance from time to time. This approach to reporting the statistical significance of their findings may have impacted their conclusion [29].

Table 3 shows an overview of the studies demonstrating weaker or conflicting evidence on PRP’s effectiveness in treating KOA.

**Table 3:** Platelet-rich-plasma (PRP): Key studies showing limited or inconclusive evidence of PRP’s effectiveness in knee osteoarthritis (KOA). This table presents an overview of studies that have investigated PRP’s efficacy in treating KOA and have reported limited or inconclusive evidence. The studies highlighted in the table have either found PRP to be effective in reducing symptoms compared to baseline (for a shorter duration) or have found no statistically significant superiority of PRP over other non-surgical treatments currently in use, emerging non-surgical treatments, or a placebo. However, some of these conclusions have lost their significance, or the trials have lost their ability to be adequately compared to the rest due to the lack of a placebo, statistical analysis, or occasional improper statistical analysis. In some instances, PRP has only been effective in improving pain or function for a single time frame. Furthermore, some clinical trials, such as the one conducted by Tucker et al. (2021) [28], may not be able to attribute the effect, or lack thereof, of PRP on patients' VAS and WOMAC scores due to a relatively smaller sample size, despite being interesting for their deep dive into the changes of the synovial environment. Note: All studies included are clinical trials that met the inclusion criteria for this review and carry a clinical evidence level of II or higher. ACS, Autologous Conditioned Serum; AS, Visual Analogue Scale; EQ-5D-5L, EuroQol Five-Dimension Five-Level Index; HA, Hyaluronic Acid; KOOS, Knee Injury and Osteoarthritis Outcome Score; mL, Milliliters; MRI, Magnetic Resonance Imaging; p, Probability; PRL, Prolotherapy; SF, Synovial Fluid; vs., Versus; WOMAC, Western Ontario and McMaster Universities Osteoarthritis Index.

Authors	Sample Size	Age (Year)	Dose/Platelet Concentration	PRP Type	Treatment Groups	Assessment Time	Assessment Tools	Findings
Bennell et al. (2021) [24]	288 patients	50 or older	5 mL dose weekly for 3 weeks at 1.6-5x the whole blood	Non-activated and leukocyte-poor	Placebo vs. PRP (144 each)	Baseline and 12 months	Pain scale 1-11 and the MRI for imaging of medial tibial cartilage volume	Intergroup analysis showed no significant difference in pain (p = 0.17) and cartilage volume (p = 0.81) between the two groups. The patients in the PRP group reported lower pain scores than baseline (no statistical comparison was available). The placebo group had a more minor loss of cartilage.
Dório et al. (2021) [25]	62 patients	45-80	1.4-5 mL dose biweekly for 2 weeks at ~3x the whole blood (standardized to 1 million platelets/cubic millimeters)	Non-activated and leukocyte-poor	PRP (20) vs. Plasma (21) vs. Saline (21)	Baseline, 6 weeks, and 12 weeks	VAS, WOMAC, and KOOS	A significant decrease was observed from baseline on VAS in all three groups. Intergroup analysis revealed that only one section of the KOOS scale, sport/recreation 0-100, demonstrated a significant decrease in the PRP group compared to the plasma and saline groups (p = 0.005).
Küçükakkaş et al. (2022) [26]	40 patients	18-80	Single injection at 7.66x the whole blood	Non-activated and leukocyte-poor	PRP (20) vs. HA (20)	Baseline, 1 month, and 6 months	VAS-rest, VAS-movement, WOMAC, Femoral Cartilage (medial and lateral) thickness	The patients in both the PRP and HA groups experienced a significant improvement in pain reduction (p < 0.05) and functional improvement (p < 0.05) compared to baseline. However, there was no significant difference between PRP and HA group. HA group showed increase in femoral cartilage thickness in the medial, lateral, and mean cartilage (p = 0.003, p < 0.001, p < 0.001, respectively). In contrast, the PRP group had no significant increase in cartilage thickness.
Pishgahi et al. (2020) [27]	92 patients	No age restriction	Dose weekly for 2 weeks at 4x the whole blood	Non-activated and leukocyte-poor	PRP (30) vs. PRL (30) vs. ACS (34)	Baseline, 1 month, and 6 months	VAS and WOMAC	The PRP group had a significant reduction in VAS scores only at the 1-month time frame (p = 0.019) when compared to the baseline. The ACS group had a significant decrease in VAS scores for both time frames, with p = 0.011 at one-month follow-up and p < 0.001 at six-month follow-up. The PRL group did not experience pain reduction throughout the study. The PRP and ACS groups showed no significant changes in WOMAC scores at the 1-month time frame. However, there was significant reduction in WOMAC scores in both PRP and ACS group at six-month follow-up (p = 0.037 and p < 0.001, respectively).
Tucker et al. (2021) [28]	16 patients	40 or older	Single 5mL injection at 2.59x the whole blood	Non-activated and leukocyte-poor	PRP (10) vs. Saline (6)	Baseline, 3 months, 6 months, and 12 months (radiographic assessment at 6 months)	VAS, WOMAC, SF analysis, and radiograph	The PRP group showed a significant reduction in stiffness after three (p < 0.05), six (p < 0.05), and 12 months (p < 0.05), and also experienced a reduction in physical function impediments after three (p < 0.05) and six months (p < 0.05). The VAS scores consistently showed a decrease in pain for the PRP group; however, the reduction was not statistically significant. A significant rise in the alpha-2-macroglobulin protein (p < 0.005) was noted in the SF of the group treated with PRP. Due to the limited sample size, the radiographic assessment did not provide adequate information to draw significant conclusions.
Lewis et al. (2022) [29]	102 patients	18 or older	Saline + PRP: single injection at 2x the whole blood PRP only: Dose weekly for 3 weeks at 2x the whole blood	Non-activated and leukocyte-poor	Saline (28) vs. Saline + PRP (47) vs. PRP (27)	Baseline, 6 weeks, 12 weeks, 26 weeks, and 52 weeks	KOOS and EQ-5D-5L	Both the saline and single-dose PRP groups showed decreased scores at all the follow-up time points (p < 0.0041). The full-dose PRP group only achieved statistical significance in the final measurement at 52 weeks (p = 0.007). The intergroup analysis only revealed a substantial decrease in scores at a single time frame, 12 weeks, and only within the full-dose PRP group. For the intragroup analysis of the EQ-5D-5L test, the saline group showed a significant decrease in scores at the 12-week measurement (p = 0.036). The single-dose PRP group recorded a significant decline in scores at the 6, 12, and 26-week measurements (p = 0.036), while the full-dose PRP group had no significant effect on scores throughout the study. Intergroup analysis of the EQ-5D-5L showed insignificant differences between both PRP groups and the saline control group throughout the study.

Potential limitations

Throughout this review, it has been noted that the clinical trials conducted to explore PRP's effects have different PRP production protocols. This leads to different platelet concentrations and PRP classifications, which substantially weakens comparability between the studies and hinders any attempt at reconciling the results of one study with the results of another. Additionally, these clinical trials only involve studies from the last five years, and their clinical relevance and comparability are likely greater when compared to those carried out when the idea of PRP as a treatment was first emerging. It is important to note that ongoing clinical trials were not involved in this review and may have attempted to address standardization issues in the PRP investigation.

Discussion

The differences in PRP formulation are a significant issue when comparing the outcomes of many of these clinical trials. Upon analyzing the methods and results of the studies chosen, it is evident that when a PRP formulation reached a platelet concentration of over four times greater than the average whole blood platelet concentration for that trial, PRP nearly always either significantly exceeded or matched the therapeutic effects found with placebo or alternative treatments in terms of improvements in KOA-related joint pain and function. The averages of the studies concluding that PRP confers clinically relevant effects, on average, have a higher platelet concentration than those studies which found no relevant results, 4.83-5.91 and 3.48-4.04, respectively.

The available studies have different assessment tools for treatment efficacy and include different patient demographics, various treatments to which they compare PRP, a placebo group or not, different dosage schedules, and measures at different time frames post-treatment. Because of these differences, the conclusions drawn from trial comparisons will not be significant. Individually, a study can offer a conclusion on PRP treatment, but it only applies to their specific experimental setup regarding all previously mentioned variables. WOMAC and VAS are commonly used to measure pain and joint function in KOA patients. They are utilized in many of these studies, allowing some standard comparisons through shared measurement modalities. These scores are pain and function indices that are valuable tools for measuring disease symptomology progression or any clinically relevant improvement in function or pain.

Similarly, some studies used other modalities to further express the effect that various interventions may have been eliciting. 6MWD, IKDC, and the LEQ index are examples of how these studies can evaluate their patients. In contrast, some studies developed pain questionnaires or functional tests to examine specific areas of interest. Interestingly, some studies included cartilage thickness, whether it be articular cartilage, medial tibial cartilage, or femoral cartilage. PRP has been touted as reducing pain and having a structurally regenerative effect on the cartilage of the knee and the synovial environment in general. These studies explore this claim via MRI or ultrasonography of the specific area chosen and have shown mixed results, with two studies claiming no effect from PRP and the other claiming statistically significant maintenance of cartilage throughout the length of the trial when compared to placebo.

Considering that the mechanism through which KOA progresses is not fully understood, and the etiology of the disease is complex and involves many different lifestyle, metabolic, and genetic factors, understanding how a KOA treatment may alter the cytokine profile and immunologic environment of the affected joint space is crucial. This offers some exciting opportunities for future studies, as elucidating the mechanism through which KOA manifests and progresses will allow for a more targeted approach to treatment. Allowing for a more direct comparison of the available therapies and measuring their effects on the disease mechanism will enable researchers to express any clinical relevancy more clearly. This will help contextualize the seemingly vague similarities between the studies in this review, as the assessment modalities used are just a measurement of changes in symptom presentation and are not representative of any actual therapeutic influence on the progression of the disease itself.

## Conclusions

Numerous clinical studies have suggested that PRP therapy is promising in treating KOA. The evidence indicates that PRP treatment, with a mean platelet concentration of 4.83 to 5.91 times higher than baseline whole blood platelet concentration, can help alleviate pain, enhance joint function, increase range of motion, and improve mobility in KOA patients. Conversely, studies with a mean platelet concentration of 3.48 to 4.04 times higher than baseline failed to exhibit statistically significant improvements. PRP therapy has been observed to slow down disease progression, thus affirming its efficacy in arresting KOA’s degenerative effects and averting further structural injury. Nevertheless, researchers need to delve deeper into the pathophysiology of KOA and investigate how PRP therapy can modulate the disease course. Future studies should also focus on determining the optimal platelet concentration levels for maximal symptom relief. The lack of standardization in PRP manufacturing protocols, including the array of experimental setups and dosing schedules used in various studies, necessitates further research to establish the specific PRP configuration that is most clinically relevant.
